# Forensic analysis using ultra-high-performance liquid chromatography–tandem mass spectrometry with solid-phase extraction of α-solanine and α-chaconine in whole blood

**DOI:** 10.1007/s11419-018-0452-7

**Published:** 2018-11-19

**Authors:** Akina Nara, Kanju Saka, Chiho Yamada, Takanori Kodama, Tetsuya Takagi

**Affiliations:** 1 Division of Legal Medicine, Faculty of Medicine, Tohoku Medical and Pharmaceutical University, 1-15-1 Fukumuro, Miyagino-ku, Sendai-shi, Miyagi 983-8536 Japan; 2 Department of Forensic Medicine, Graduate School of Medicine, The University of Tokyo, 7-3-1 Hongo, Bunkyo-ku, Tokyo, 113-0033 Japan

**Keywords:** Glycoalkaloid, α-Solanine, α-Chaconine, Solid-phase extraction, Validation, Liquid chromatography–mass spectrometry

## Abstract

**Purpose:**

The potato glycoalkaloids (PGAs), α-solanine and α-chaconine can exert adverse effects on human health when consumed in excess. This study aimed to investigate the optimal extraction method for the quantitative analysis of PGAs in whole blood by using ultra-high-performance liquid chromatography–tandem mass spectrometry (UHPLC–MS/MS) and to apply this validated method to postmortem blood.

**Methods:**

A total of 200 µL of human whole blood was prepared for PGA extraction. For validation, a solid-phase extraction (SPE) using Oasis^®^ PRiME HLB, in which extraction could be performed in three simple steps (sample loading, washing, and elution) was used, with no need for both conditioning and equilibration of columns for sample preparation.

**Results:**

In this method, the limit of detection and the lower limit of quantification (LLOQ) of both α-solanine and α-chaconine were 1 and 2 µg/L, respectively. The calibration curves of the two compounds were obtained with good linearity in the range of 2–100 µg/L. The recovery rates at the LLOQ of α-solanine and α-chaconine were ≥ 91.8% and ≥ 85.9%, respectively. The validation data (intra- and inter-day combined) for accuracy ranged from 93.5 to 106.6% for α-solanine and from 93.9 to 107.7% for α-chaconine. This validated method was successfully applied to one forensic autopsy case, and the concentrations of α-solanine and α-chaconine in the postmortem cardiac blood were 45.1 and 35.5 µg/L, respectively.

**Conclusions:**

This validated UHPLC–MS/MS with SPE for quantitative analysis of PGAs could be useful in forensic toxicology.

**Electronic supplementary material:**

The online version of this article (10.1007/s11419-018-0452-7) contains supplementary material, which is available to authorized users.

## Introduction

**Fig. 1:**
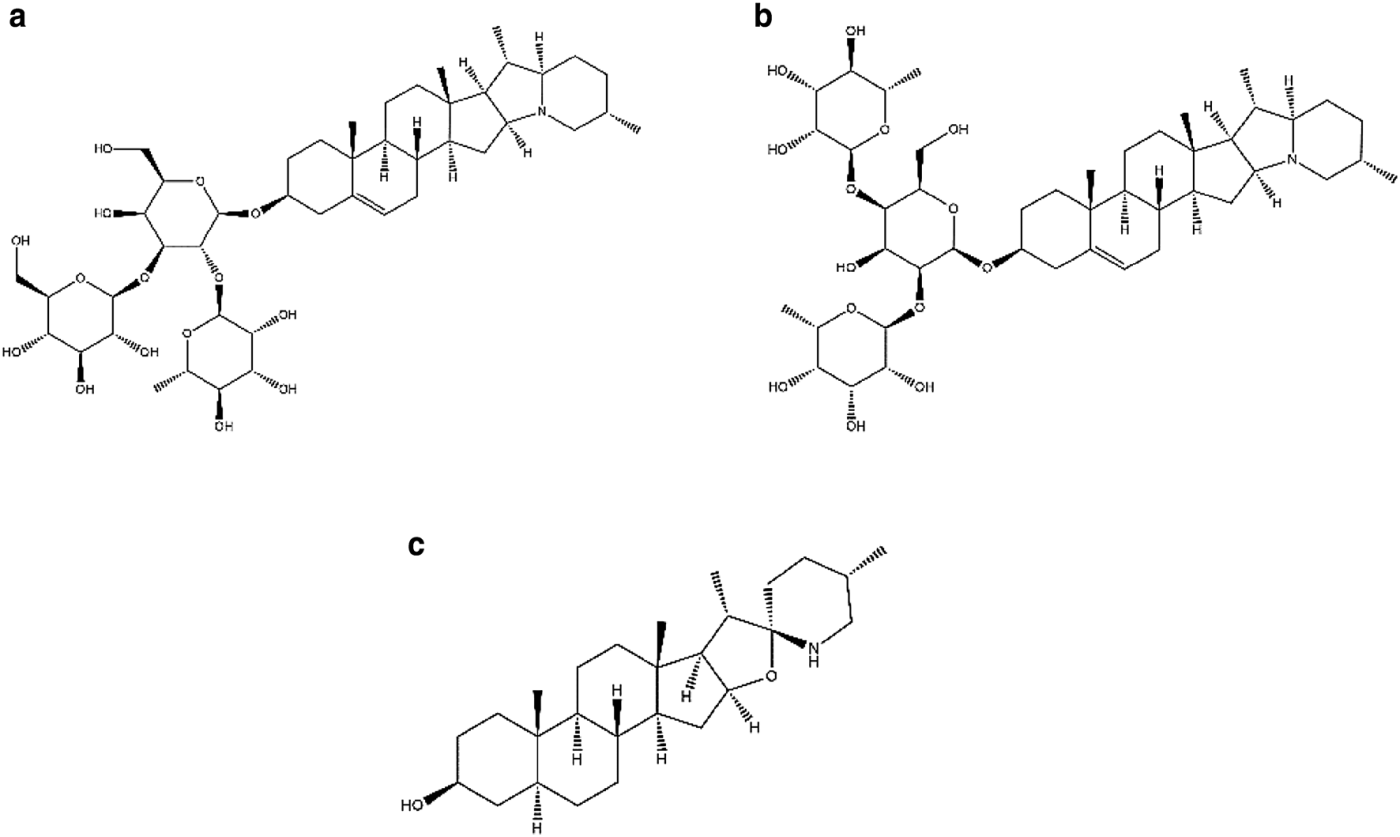
Chemical structures of α-solanine (**a**), α-chaconine (**b**), and tomatidine (**c**)

Potato (*Solanum tuberosum L*.) is a common staple food in the human diet [1]. There are two major potato glycoalkaloids (PGAs), α-solanine and α-chaconine (Fig. 1), which constitute approximately 95% of the glycoalkaloids (GAs) in potato tubers [2, 3]. The ratio of α-solanine to α-chaconine in potato tubers is about 2:3 [3], and the PGA content of peel is higher than that of the tuber flesh, especially for α-chaconine [4–6]. Generally, the PGA content of most commercial potatoes does not exceed 10 mg/100 g [3]. The maximum acceptable PGA content has been set at 20–25 mg/100 g of fresh potato weight [7]. However, the concentrations of PGAs increase upon exposure to sunlight, with the potato peel beginning to turn green [3, 8, 9]. A clinical trial with human taste panels showed that potatoes with GA contents exceeding 14 mg/100 g tasted bitter and those exceeding 22 mg/100 g produced a mild to severe burning sensation in the mouth and throat [10]. PGAs have some toxic effects on mammals [5, 11, 12]. They cause gastrointestinal and systemic effects by disrupting the cell membrane and inhibiting the activity of the enzyme acetylcholinesterase in humans [1, 3, 4]. Toxic effects of ingesting PGAs are induced in humans within several minutes to several days after consuming potatoes [11, 13, 14]. The intake of potatoes rich in GAs can result in symptoms such as nausea, vomiting, and abdominal pain [13, 14]. Since the clearance of PGAs usually takes more than 24 h [11], it has been suggested that accumulation of PGAs associated with long-term excessive consumption of potatoes has adverse effects on human health. PGA poisoning cases involving ingestion of potatoes with dangerous amounts of PGAs have been reported over the decades [13–16]. In Japan, food poisoning data for 50 years indicated that there were 23 incidents of PGA poisoning involving 918 people [17]. Occasionally, PGA poisoning can be fatal. Indeed, some autopsy cases of lethality associated with the ingestion of PGAs have been reported [15, 16]. Therefore, in forensic toxicology, it is quite important to develop an analytical procedure for the detection of PGAs that can be used for analysis of postmortem specimens.

For the validation method of PGAs contained in fresh and processed potatoes, high-performance liquid chromatography–tandem mass spectrometry (HPLC–MS/MS) or ultra-HPLC–tandem mass spectrometry (UHPLC–MS/MS) have been commonly used [18–22]. There have also been some research reports of an HPLC method for analyzing PGAs in human serum and of an UHPLC–MS/MS screening method for measuring 34 toxic compounds of plant origin, including α-solanine (not including α-chaconine), in human whole blood [23, 24]. These analytical methods for the detection of PGAs in human samples have used solid-phase extraction (SPE), a sample preparation technique often used to isolate selected analytes. SPE methods have often been used for the processing of human blood, serum, and plasma samples in forensic toxicology [25, 26]. The sensitivity and selectivity of SPE followed by HPLC–MS/MS or UHPLC–MS/MS have been improved by advancements in analytical instruments and techniques; however, sample matrix-induced ion suppression or enhancement, known as the matrix effect, is one of the problems associated with an electrospray ionization (ESI) source [27–31]. Additionally, in SPE methods, the analyte sometimes remains predominantly bound to the sample matrix because of incomplete partitioning into the stationary phase, resulting in low analyte recovery [25]. Therefore, it is necessary to establish an optimal SPE method for PGAs that can reduce the matrix effect and increase the recovery.

In this study, we developed an optimal SPE with the UHPLC–MS/MS method that can be simply performed for the determination of PGAs in human whole blood and used it for quantitative analysis in forensic autopsy cases in which PGA poisoning is suspected.

## Materials and methods

### Chemicals and reagents

α-Solanine, α-chaconine, and tomatidine (Fig. 1) were obtained from Sigma-Aldrich (St. Louis, MO, USA). Ammonium formate (analytical grade), formic acid (LC/MS grade), methanol (LC/MS grade), and ultrapure water (LC/MS grade) were purchased from FUJIFILM Wako Pure Chemical Corporation (Osaka, Japan). Oasis^®^ PRiME HLB cartridges were purchased from Waters (Milford, MA, USA). Millex^®^ LH syringe filters were purchased from Merck Millipore (Burlington, MA, USA).

### Preparation of quality control samples and calibration standards

Stock solutions (1 g/L) of α-solanine, α-chaconine, and tomatidine were prepared in methanol. All the solutions were stored at −20 °C in the dark when not used. Working standard solutions were prepared by diluting the stock solutions with methanol and were stored at 4 °C. A 100 µg/L solution of tomatidine in methanol was used as the internal standard (IS) for measurement of α-solanine and α-chaconine. Blank whole blood was collected from healthy volunteers after they had provided informed consent, and the samples were screened for α-solanine, α-chaconine, and tomatidine, all of which were confirmed to be negative.

Calibration curves were prepared by using spiked whole blood (200 µL) with appropriate volumes of the working standard solutions for six points equivalent to 2, 5, 10, 20, 50, and 100 µg/L α-solanine and α-chaconine, and IS was also spiked into the whole blood as 10 µg/L. Quality control (QC) samples at 2 (lower limit of quantification; LLOQ), 8 (low), 40 (medium), and 80 (high) µg/L for α-solanine and α-chaconine were prepared in bulk by spiking the appropriate working standard solutions into whole blood (200 µL).

### Sample preparation

SPE of α-solanine and α-chaconine were performed by using an Oasis^®^ PRiME HLB in which both conditioning of the column and the equilibration step can be omitted and sample preparation is performed quickly and simply [32, 33]. SPE using this cartridge consisted of three steps (sample loading, washing, and elution). The sample (200 µL of whole blood), which was mixed with 20 µL of 100 µg/L IS solution and 400 µL of ultrapure water, was directly applied to the cartridge. The column was rinsed with 3 mL of 30% methanol and allowed to drain under reduced pressure using a GL-SPE vacuum manifold system (GL Sciences, Tokyo, Japan) for 1 min. Then, elution was performed using 1 mL of 100% methanol. The eluate was subsequently evaporated to dryness under a stream of N_2_ gas at 45 °C, and the residue was reconstituted in 200 µL of 10 mM ammonium formate with 0.1% formic acid in methanol (mobile phase B) and then centrifuged at 12,000*g* for 5 min. The supernatant was filtered through Millex^®^ LH syringe filters (pore size, 0.45 µm), and 5 µL of the filtrate was injected into the UHPLC–MS/MS system.

### UHPLC–MS/MS conditions

Qualitative and quantitative analyses were performed by using a Nexera X2 HPLC system coupled with a Shimadzu LCMS-8045 triple quadrupole mass spectrometer (Shimadzu, Kyoto, Japan). Chromatographic separation was achieved by using a Kinetex^®^ XB-C18 column (100 × 2.1 mm i.d.; particle size, 2.6 µm; Phenomenex, Torrance, CA, USA) with Security Guard ULTRA cartridge system (UHPLC C18 for 2.1 mm ID column; Phenomenex) maintained at 40 °C. The mobile phase consisted of 10 mM ammonium formate with 0.1% formic acid in water (A) and in methanol (B). The flow rate was 0.4 mL/min. The gradient program was as follows: 5–95% B from 0 to 7 min and then 95% B until 8.5 min. At 8.6 min, the concentration of B returned to 5% and remained constant until 10 min.

The mass spectrometer was operated in positive mode, with an ESI interface. The ionization source conditions were as follows: nebulizer gas flow rate of 3 L/min, heating gas flow rate of 10 L/min, drying gas flow rate of 10 L/min, interface temperature of 300 °C, desolvation line temperature of 250 °C, and heat block temperature of 400 °C. The collision gas was high-purity argon, and the nebulizer gas was nitrogen. Analytes were detected by using the multiple reaction monitoring (MRM) mode. In the MRM transitions, two product ions (*m/z*), one used as a quantifier and the other as a qualifier, were monitored for each compound (Table 1). The product ions and collision energy were determined by post-column infusion of a methanol solution of each compound (Table 1). Labsolutions Insight Ver. 3.10 SP1 software (Shimadzu) was used to perform the quantitative analysis of all the data.

**Table 1:** Retention time, precursor ion, product ion and collision energy of α-solanine, α-chaconine, and IS

Compound	Retention time (min)	Precursor ion (*m/z*)	Product ion^a^ (*m/z*)	Collision energy (eV)
α-Solanine	5.08	868.40	**398.05**	75.5
			98.05	86.5
α-Chaconine	5.08	852.40	**706.40**	72.5
			98.10	86.1
Tomatidine (IS)	6.42	416.40	**255.30**	33.0
			161.05	39.0

*IS* internal standard

^a^Bold type is used for quantifiers and normal type for qualifiers

### Validation of the method

The validation of the method was performed with reference to US food and drug administration (FDA) guidance [34]. The method was validated by establishing selectivity, linearity, limit of detection (LOD), LLOQ, matrix effect, recovery, intra- and inter-day accuracy and precision, and stability. The selectivity of the method was measured by comparing the chromatograms of blank human whole blood of six different origins to ensure no interference at the retention times of α-solanine, α-chaconine, and the IS from blood. The linearity of this method was expressed as a correlation coefficient (*r*) of the standard curve. The LOD and LLOQ, which corresponded to signal/noise ratios of ≥ 3 and ≥ 10, respectively, were calculated as the concentrations of analytes in QC samples. Validation data (matrix effect, recovery, and stability) are expressed as the mean ± standard deviation (S.D.).

The matrix effect and recovery for α-solanine and α-chaconine were evaluated by measuring the peak area of four QC concentrations (2, 8, 40, and 80 µg/L). Six different experiments were performed for the determination of the matrix effects and recovery. The matrix effect was determined by comparison of the peak area of α-solanine or α-chaconine extracted from whole blood samples spiked with working solutions, after the extraction with that of a neat solution without this extraction. Recovery was determined by comparison of the peak area of α-solanine or α-chaconine extracted from whole blood samples spiked with working solutions before extraction with the peak area of the compound spiked after extraction. The precision of the recovery was defined as the ratio of the S.D. of a set of data to the mean expressed as a percentage at each QC sample concentration.

QC samples at four concentrations (2, 8, 40, and 80 µg/L) of α-solanine and α-chaconine were analyzed over 3 days to determine the intra- and inter-day accuracy and precision. Intra-day accuracy and precision were determined by six replicate analyses at four QC concentrations on the calibration curves. Inter-day accuracy and precision of QC samples were obtained by three replications of the intra-day assay. The accuracy of quantification for the QC samples at each set of concentrations was determined from the calculated concentrations obtained from the standard curve. Precision was calculated as the percentage of the coefficient of variation (CV) of the replicates at each of the concentrations in the inter- and intra-day analyses. According to the FDA guidance, accuracy and precision should be within ± 15% except at the LLOQ, at which the accuracy and precision should be within ± 20% [34].

To evaluate the stabilities of short- and long-term storage or freeze–thaw cycles in this method, three QC samples of α-solanine and α-chaconine at four concentrations (2, 8, 40, and 80 µg/L) were either stored at room temperature for 24 h, at 4 °C for 7 days, and at −20 °C for 4 weeks or subjected to three freeze–thaw cycles. The stability of each QC sample concentration was determined by expressing the mean concentrations obtained from the standard curve as percentages of the known concentrations. The stability of the samples only had to be between 85% and 115%.

### Case report

A male in his early 60s had been living in his car. He was found dead in the car approximately 1 month after he had last been seen alive. A police officer stated that he frequently consumed potatoes in the car. On external examination, the man was 178 cm in height and 69.2 kg in weight. Toxicological evaluation of postmortem cardiac blood was performed by using the LC/MS/MS rapid toxicology screening system ver. 2 (Shimadzu); drugs that could have directly caused his death were not detected. For the qualitative and quantitative analyses of α-solanine and α-chaconine, the validated method described in the present report was used.

## Results and discussion

### Method development

Our study aim was to establish an optimal method for analyzing PGAs in human whole blood for use in the field of forensic toxicology. SPE methods are often used in forensic laboratories to extract drugs from postmortem blood [26]. In an SPE method, expected concentrations in biological samples may be much higher and therefore a smaller sample size should be used [25]. Additionally, ineffective retention of target analytes on the solid-phase sorbent could be avoided by decreasing the sample size [25]. Therefore, in the present study, 200 µL of human whole blood was used for sample preparation to perform a simple SPE method.

In various SPE separation mechanisms, “reversed-phase” has been commonly used for the extraction of drugs and other substances from various matrices [25]. Oasis^®^ HLB (Waters), which is one of the cartridges used in the SPE method, is known as a polymeric reversed-phase sorbent for extraction of a wide range of acidic, basic, and neutral compounds from various matrices [35, 36]. High recovery rates (~ 100%) of standard PGAs have been obtained with this cartridge [37]. However, before applying samples to the cartridge, it is essential to condition these columns with methanol followed by water. On the other hand, compared with Oasis^®^ HLB, Oasis^®^ PRiME HLB, which can also be used for reversed-phase cleanup of acidic, basic, and neutral compounds from complex sample matrices, has been designed to simplify SPE with easy-to-follow protocols [33]. With this cartridge, it is possible to apply the samples (e.g., whole blood) directly without the need for both conditioning of the column and the equilibration step. Recently, a microelution-SPE HPLC–MS/MS method using this cartridge for the quantitative analysis of atypical antipsychotic drugs in human plasma was demonstrated [32]. Although endogenous phospholipids in human plasma tend to cause an ion suppression or enhancement, the Wojnicz et al. method [32] successfully removed ≥ 99% of endogenous plasma phospholipids, which was superior to that for protein precipitation with acetonitrile. Therefore, we selected this cartridge as being suitable for quick and simple sample preparation in the SPE method.

For the preliminary experiment, we determined the optimal extraction method using Oasis^®^ PRiME HLB for the sample preparation. For sample loading, 200 µL of whole blood with IS solution was mixed with 200 µL of ultrapure water; however, it took quite some time to pass through the column. In another experiment, the sample was mixed with 400 µL of ultrapure water, then the sample passed quickly. In a previously reported, validated method for detecting α-solanine [23], the washing step in SPE using Oasis^®^ HLB was performed using three volumes of 1 mL of deionized water; accordingly, we first used ultrapure water in the washing step, although we later used another cartridge, Oasis^®^ PRiME HLB. The mean values of the recovery of both α-solanine and α-chaconine at four QC concentrations (2, 8, 40, and 80 µg/L) were both ≥ 90% (*n* = 6) (Table S1). The mean values of the matrix effects of α-solanine and α-chaconine at the four QC concentrations (2, 8, 40, and 80 µg/L) were 129.0–221.8% and 89.3–128.2%, respectively(*n* = 6). These higher values of the matrix effect may have been due to matrix-induced ion enhancement [38]. Conversely, Sep-Pak C18 cartridges (Waters) were used as SPE cartridges for measuring α-solanine and α-chaconine concentrations in potatoes, and during the washing step, 30% methanol was used [18, 39]. Therefore, in the washing steps, 30% of methanol was tested using the Oasis^®^ PRiME HLB cartridge. The matrix effects of α-solanine and α-chaconine (detailed data are shown in “Method validation”) were relatively good compared with those obtained using washing with ultrapure water.

### UHPLC–MS/MS procedure

**Fig. 2:**
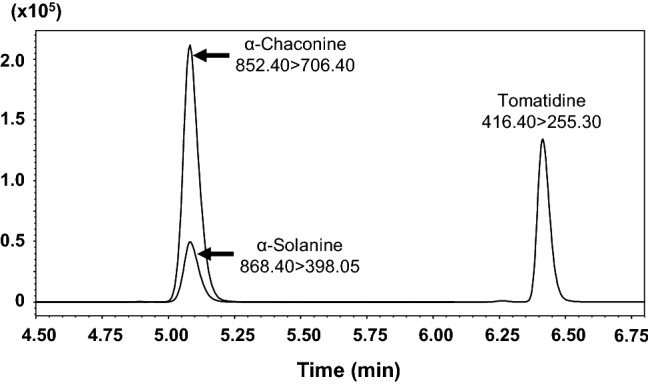
Multiple reaction monitoring chromatograms of α-solanine, α-chaconine, and tomatidine of human whole blood spiked with 100 µg/L

**Fig. 3:**
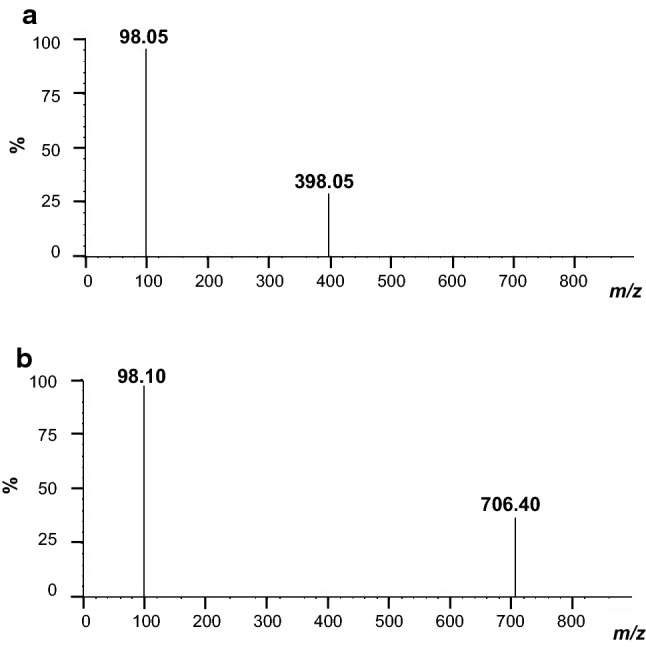
Product ion spectra obtained from fragmentation of α-solanine (**a**) and α-chaconine (**b**) in human whole blood

The positive and negative detection modes were tested during the infusion of α-solanine and α-chaconine. Positive mode was more appropriate than negative mode for the compounds because it provided a better signal/noise ratio. The [M+H]^+^ adduct was selected as a precursor ion for α-solanine, α-chaconine, and IS (Table 1). The separation column (Kinetex^®^ XB-C18) used in this method was able to analyze α-solanine, α-chaconine, and IS quickly; furthermore the compounds showed good peak shape (Fig. 2). As shown in Fig. 2, the peaks of α-solanine and α-chaconine overlapped in the gradient program with a total run of 10 min. Furthermore, the peaks did not resolve even following extension of run time of the gradient program to 15 or 20 min (Figure S1). However, α-solanine and α-chaconine were sufficiently identified, as assessed using the product ion spectra in MRM (Fig. 3); therefore, we adopted the gradient program with a shorter run time (10 min). Under the chromatographic conditions used, there was no interference with the analytes by any extractable endogenous materials present in whole blood. On the basis of these results, this SPE was performed on an Oasis^®^ PRiME HLB cartridge for validation of quantification of α-solanine and α-chaconine in human whole blood.

### Method validation

Table 2 presents the calibration curve, correlation efficient, concentration range, LOD, and LLOQ of α-solanine and α-chaconine. The calibration curve consisted of blank whole blood with IS at six concentrations (2, 5, 10, 20, 50, and 100 µg/L), including the LLOQ. The peak-area ratios of the target compounds and their respective IS were calculated for each standard curve. The LOD values of α-solanine and α-chaconine were determined as the lowest concentration giving a response three times the level of a blank response. The LLOQ values of α-solanine and α-chaconine were set as the concentrations with a response 10 times higher than those of the blank matrix samples. Calibration curves of α-solanine and α-chaconine up to 100 µg/L were produced, and the results of the mean values were ≤ 20% of the LLOQ from the expected concentration and ≤ 15% of the higher concentrations from their expected concentrations. Good linearity was shown (*r* > 0.999) in the calibration curves of the two compounds. Overall, the results obtained by using this method with whole blood were linear and sensitive for each analyte.

**Table 2:** Calibration curve, correlation coefficient, concentration range, LOD and LLOQ of α-solanine and α-chaconine

Compound	Calibration curve^a^	Correlation coefficient (*r*)	Concentration range (µg/L)	LOD (µg/L)	LLOQ (µg/L)
α-Solanine	*y* = 0.0352*x*–0.00298	0.999	2–100	1	2
α-Chaconine	*y* = 0.161*x*–0.00679	0.999	2–100	1	2

*LOD* limit of detection, *LLOQ* lower limit of quantification

^a^In the equation of the calibration curve, “*x*” denoted the concentration ratio of α-solanine (or α-chaconine) to that of tomatidine and “*y*” denoted the peak area ratio of α-solanine (or α-chaconine) to that of tomatidine

Table 3 summarizes the results showing the matrix effect and from the recovery examinations with human whole blood. Carlier et al. demonstrated that the percentage of the matrix effect at 100 µg/L α-solanine in human whole blood using Oasis^®^ HLB was 73% [23]. In our study, the mean values of the matrix effect of α-solanine and α-chaconine at four QC concentrations ranged from 83.6 to 107.9% and from 87.6 to 106.7%, respectively. In the FDA guidance, the recovery of analytes need not reach 100% but should be consistent, precise, and reproducible [34]. The studied method gave recoveries of ≥ 80% at all QC concentrations for both α-solanine and α-chaconine. The peaks corresponding to analytes, which were collected at each step of sample loading, washing, and elution in the SPE method (ultrapure water was used instead of whole blood), were not detected during both sample loading and washing, whereas these peaks were sufficiently detected during elution (Figure S2). The remaining analytes that were not recovered in this SPE method were assumed not to have eluted through the column and were assumed to have been absorbed by it. Additionally, the precision of the recovery at all QC concentrations of the two compounds was ≤ 6.9%. Therefore, our method could be accurately applied for the sample preparation of α-solanine and α-chaconine from whole blood by overcoming the problems related to recovery in the SPE method.

**Table 3:** Matrix effect and recovery of α-solanine and α-chaconine in the whole blood at four QC concentrations (2, 8, 40, and 80 µg/L)

Compound	QC concentration (µg/L)	Matrix effect^a^ (%)	Recovery^b^ (%)
α-Solanine	2 (LLOQ)	107.9 ± 3.5	98.6 ± 6.8
	8 (low)	93.3 ± 3.3	103.9 ± 6.2
	40 (medium)	88.4 ± 4.6	98.8 ± 3.2
	80 (high)	83.6 ± 4.2	95.2 ± 3.1
α-Chaconine	2 (LLOQ)	106.5 ± 3.5	90.2 ± 4.3
	8 (low)	87.6 ± 0.7	97.3 ± 2.8
	40 (medium)	104.0 ± 4.6	83.8 ± 2.2
	80 (high)	106.7 ± 12.1	84.2 ± 1.3

*LLOQ* lower limit of quantification

^a^Data are expressed as the ratio (%) with mean ±  standard deviation of the peak area of the extracts spiked with working solutions after the extraction relative to the peak area of the neat solutions

^b^Data are presented as the mean ± standard deviation

For the validation data (intra- and inter-day combined) at all QC concentrations, accuracy ranged from 93.5 to 106.6% for α-solanine and from 93.9 to 107.7% for α-chaconine (Table 4). The accuracies of the medium and high concentrations for α-solanine and α-chaconine tended to be closer to 100% than the accuracies for the LLOQ and low concentrations. The CV (%) data (intra- and inter-day combined) of α-solanine and α-chaconine were ≤ 10% (Table 4), and these validated data met the criteria indicated in the FDA guidance [34]. There were no significant differences in accuracy and precision at each concentration of the QC samples between the intra- and inter-day data. These results indicated that this method gave satisfactory accuracy and precision for α-solanine and α-chaconine obtained from whole blood. Furthermore, we confirmed the validity of the calibration curve in the range of 2–100 µg/L of α-solanine and α-chaconine using the whole blood of 1000 µg/L of these analytes, which were diluted to 10-fold during sample preparation. The accuracy of the method for quantifying α-solanine and α-chaconine in these samples was 103.3% and 107.1% and the precision was 1.7% and 3.4%, respectively (*n* = 6). Therefore, the calibration curves could be extended from 2 to 1000 µg/L using appropriate dilution.

**Table 4:** Intra- and inter-day accuracy and precision of α-solanine and α-chaconine in the whole blood at four QC concentrations (2, 8, 40, and 80 µg/L)

Compound	QC concentration (µg/L)	Intra-day		Inter-day	
		Accuracy (%)	CV (%)	Accuracy (%)	CV (%)
α-Solanine	2 (LLOQ)	95.9	4.8	106.6	6.8
	8 (low)	96.3	9.4	93.5	5.7
	40 (medium)	103.0	3.5	99.6	2.9
	80 (high)	101.1	3.1	101.6	2.0
α-Chaconine	2 (LLOQ)	99.4	3.3	107.6	3.6
	8 (low)	93.9	1.7	97.6	1.5
	40 (medium)	100.5	2.6	98.2	1.3
	80 (high)	99.5	1.3	101.0	1.6

*QC* quality control, *LLOQ* lower limit of quantification, *CV* coefficient of variation

Table 5 summarizes the results of the stability assays of four QC samples. The ratio (%) of the measured concentrations of α-solanine and α-chaconine to the theoretical concentrations ranged from 85.1 to 104.2% after storage at room temperature for 24 h, at 4 °C for 7 days, and at −20 °C for 4 weeks and ranged from 85.9 to 102.8%, after being subjected to three freeze–thaw cycles. The stability of the two compounds before analysis had little effect on the quantification. One report on a stability experiment using the SPE method for PGAs in human serum noted that the concentrations when the two compounds were initially set at 2, 10, 25, and 100 µg/L were all stable during test periods of 24 h at 37 °C and 2 months at −20 °C [24]. Our results in this method certified the stability of the PGAs during the storage conditions.

**Table 5:** The stabilities of α-solanine and α-chaconine in the whole blood after 24 h at room temperature, 7 days at 4 °C, 4 weeks at −20 °C, and three freeze–thaw cycles

Compound	QC correlation (µg/L)	RT for 24 h		4 °C for 7 days		− 20 °C for 4 weeks		3 Freeze–thaw cycles	
		Mean ± S.D.(µg/L)	Stability (%)^a^	Mean ± S.D.(µg/L)	Stability (%)^a^	Mean ± S.D.(µg/L)	Stability (%)^a^	Mean ± S.D.(µg/L)	Stability (%)^a^
α-Solanine	2 (LLOQ)	1.79 ± 0.12	89.5	1.93 ± 0.10	96.6	2.02 ± 0.11	101.1	2.06 ± 0.05	102.8
	8 (Low)	6.84 ± 0.95	85.5	6.84 ± 0.44	85.5	6.84 ± 0.43	85.5	6.87 ± 0.26	85.9
	40 (medium)	40.5 ± 0.64	101.1	34.8 ± 0.88	87.1	41.1 ± 0.67	102.7	35.6 ± 1.27	89.0
	80 (high)	83.4 ± 0.30	104.2	73.4 ± 1.44	91.8	79.7 ± 0.42	99.6	78.7 ± 1.03	98.4
α-Chaconine	2 (LLOQ)	1.72 ± 0.04	85.8	1.7 ± 0.04	85.1	1.8 ± 0.07	89.8	1.76 ± 0.08	88.0
	8 (low)	7.29 ± 0.03	91.1	7.51 ± 0.15	93.9	7.49 ± 0.27	93.6	7.04 ± 0.31	88.0
	40 (medium)	39.4 ± 0.26	98.1	37.0 ± 0.58	92.6	39.6 ± 0.18	98.9	35.4 ± 0.38	88.5
	80 (high)	79.8 ± 0.68	99.8	72.1 ± 0.74	90.1	78.7 ± 0.22	98.3	76.5 ± 2.77	95.6

*QC* quality control, *LLOQ* lower limit of quantification, *RT* room temperature, *S.D.* standard deviation

^a^Data are expressed as the ratio (%) of the measured concentrations to the theoretical concentrations

### Forensic application

**Fig. 4:**
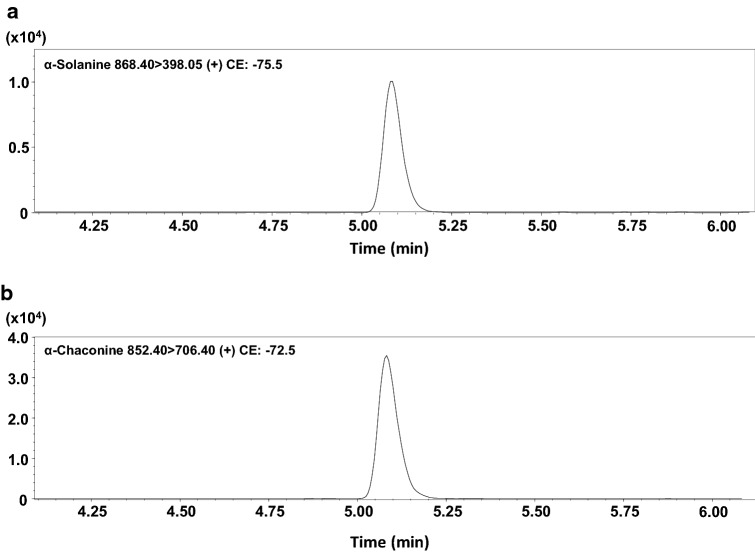
Chromatograms of α-solanine (**a**) and α-chaconine (**b**) found in the cardiac blood of this case

The concentrations of α-solanine and α-chaconine in the postmortem cardiac blood were 45.1 and 35.5 µg/L, respectively (Fig. 4).

Generally, the intake of 3–6 mg PGAs/kg body weight (BW) is considered a potentially lethal dose for humans [40]. In a clinical trial to evaluate the acute toxic effect of PGAs on humans, one of the subjects who ate mashed potatoes containing a PGA dose of 1.25 mg/kg BW experienced clinical features of PGA poisoning including gastrointestinal effects such as nausea and vomiting 4 h after the dose [11]. Since the elimination half-lives of α-solanine and α-chaconine are 21 and 44 h, respectively, on average [11], daily consumption of potatoes may lead to accumulation of these molecules in the body and may result in toxicity. PGA poisoning cases have been reported over the decades [13–16]. In a case of fatal solanine poisoning, 7 mg of solanine was detected in approximately one-third of the liver [15]. In addition, in a case of PGA poisoning of a man who died after ingestion of potato soup, fulminant hepatitis was caused by the ingestion of a severe hepatotoxic agent, which was presumed to have been solanine [41]. To the best of our knowledge, neither research reports nor forensic autopsy case reports describing toxic blood concentrations of PGAs in humans have been published. Furthermore, because of the advanced decomposition of the corpse in our case, we could not determine if fatal PGA poisoning due to the ingestion of potatoes had occurred.

## Conclusions

Quantitative analyses of PGAs performed by using the UHPLC–MS/MS with SPE method described, validated, and clinically applied in this study can provide useful information in forensic toxicology investigations.

## Electronic supplementary material

Below is the link to the electronic supplementary material.

Supplementary material 1 (DOCX 55 kb)
