# Supplementary material for: Forensic analysis using ultra-high-performance liquid chromatography–tandem mass spectrometry with solid-phase extraction of α-solanine and α-chaconine in whole blood
Source: Forensic Toxicol. 2018 Nov 19;37(1):197–206. doi: 10.1007/s11419-018-0452-7 (PMC6314998; doi:10.1007/s11419-018-0452-7)
Supplement: Supplementary file 1 — Supplementary material 1 (DOCX 55 kb) [file 11419_2018_452_MOESM1_ESM.docx]

**Forensic analysis using ultra-high-performance liquid chromatography–tandem mass spectrometry with solid-phase extraction of α-solanine and α-chaconine in whole blood**

Akina Nara ^1^, Kanju Saka ^2^, Chiho Yamada ^1^, Takanori Kodama ^1^, and Tetsuya Takagi ^1^.

^1^ Division of Legal Medicine, Faculty of Medicine, Tohoku Medical and Pharmaceutical University, 1-15-1 Fukumuro, Miyagino-ku, Sendai-shi, Miyagi 983-8536, Japan.

^2^ Department of Forensic Medicine, Graduate School of Medicine, The University of Tokyo, 7-3-1 Hongo, Bunkyo-ku, Tokyo 113-0033, Japan.

E-mail address: [akina@tohoku-mpu.ac.jp](mailto:akina.legm@gmail.com)

**Supplementary Material**

Table S1 Matrix effect and recovery of α-solanine and α-chaconine in the whole blood at four QC concentrations (2, 8, 40, and 80 µg/L), performed using 3 mL of ultrapure water at the washing step during extraction.

Figure S1 Multiple reaction monitoring chromatograms of α-solanine, α-chaconine, and tomatidine using a 100-µg/L methanol solution with a total run time of 15 min (a) and 20 min (b).

Figure S2 Multiple reaction monitoring chromatograms of extracts of 100 µg/L of α-solanine, α-chaconine, and tomatidine in ultrapure water, which were collected at each step of sample loading (a), washing (b), and elution (c) in the SPE method.


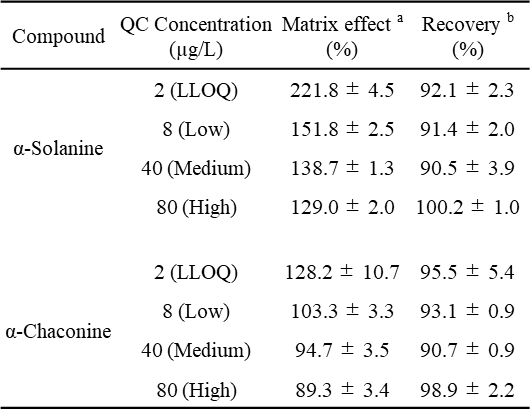


Table S1 Matrix effect and recovery of α-solanine and α-chaconine in the whole blood at four QC concentrations (2, 8, 40, and 80 µg/L), performed using 3 mL of ultrapure water at the washing step during extraction (*n* = 6).

^a^ Data are expressed as the ratio (%) with mean ± standard deviation of the peak area of the extracts spiked with working solutions after the extraction relative to the peak area of the neat solutions.

^b^ Data are presented as the mean ± standard deviation.

*LLOQ* lower limit of quantification


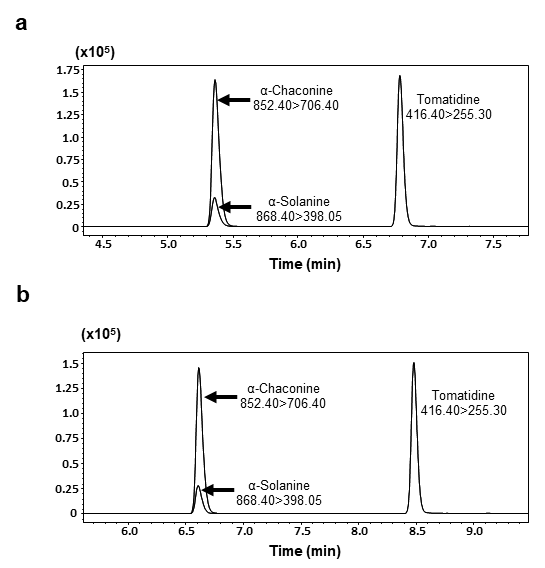


Figure S1 Multiple reaction monitoring chromatograms of α-solanine, α-chaconine, and tomatidine using a 100-µg/L methanol solution with a total run time of 15 min (a) and 20 min (b).


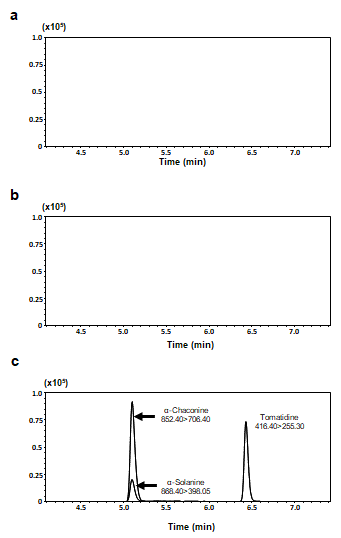


Figure S2 Multiple reaction monitoring chromatograms of extracts of 100 µg/L of α-solanine, α-chaconine, and tomatidine in ultrapure water, which were collected at each step of sample loading (a), washing (b), and elution (c) in the SPE method.
